# Plant–herbivore interactions: Experimental demonstration of genetic variability in plant–plant signalling

**DOI:** 10.1111/eva.13531

**Published:** 2023-03-29

**Authors:** Aurélien Estarague, Cyrille Violle, Denis Vile, Anaïs Hany, Thibault Martino, Pierre Moulin, François Vasseur

**Affiliations:** ^1^ CEFE, Univ Montpellier, CNRS, EPHE, IRD Montpellier France; ^2^ LEPSE, Univ Montpellier, INRAE, Institut Agro Montpellier France

**Keywords:** *Arabidopsis thaliana*, *Cornu aspersum*, genome‐wide association study, herbivory, intraspecific diversity

## Abstract

Plant–herbivore interactions mediated by plant–plant signalling have been documented in different species but its within‐species variability has hardly been quantified. Here, we tested if herbivore foraging activity on plants was influenced by a prior contact with a damaged plant and if the effect of such plant–plant signalling was variable across 113 natural genotypes of *Arabidopsis thaliana*. We filmed the activity of the generalist herbivore *Cornu aspersum* during 1 h on two plants differing only in a prior contact with a damaged plant or not. We recorded each snails' first choice, and measured its first duration on a plant, the proportion of time spent on both plants and leaf consumption. Overall, plant–plant signalling modified the foraging activity of herbivores in *A. thaliana*. On average, snails spent more time and consumed more of plants that experienced a prior contact with a damaged plant. However, the effects of plant–plant signalling on snail behaviour was variable: depending on genotype identity, plant–plant signalling made undamaged plants more repellant or attractive to snails. Genome‐wide associations revealed that genes related to stress coping ability and jasmonate pathway were associated to this variation. Together, our findings highlight the adaptive significance of plant–plant signalling for plant–herbivore interactions.

## 1 INTRODUCTION

Plants adjust their defences in response to cues from neighbours that are being attacked by herbivores (Agrawal, 2000; Bouwmeester et al., 2019; Heil & Adame‐Álvarez, 2010; Heil & Karban, 2010; Karban et al., 2000, 2003; Moreira & Abdala‐Roberts, 2019; Moreira et al., 2018). Interactions between damaged plants and their neighbours are generally associated with increased levels of plant defence. This phenomenon appears widespread across biomes, growth forms and phylogeny (Karban, 2021; Karban et al., 2014). The effect of plant–plant signalling on plant–herbivore interactions is often considered adaptive, as it is expected to be naturally selected to reduce plant biomass loss, leading to increased individual fitness (Heil & Karban, 2010). However, the extent to which this process is genetically variable, which is a prerequisite for natural selection to act (Falconer et al., 1996), has hardly been examined so far.

Plant–plant signalling can have prompt effects on herbivore foraging activity. Contact with a damaged plant can prime defences, which allows faster response in case of future herbivore attack (Engelberth et al., 2004; Heil & Bueno, 2007; Zhang et al., 2020). Plants that receive herbivore‐induced signals emitted by a neighbour can also directly express volatile molecules, that constitute a distant and repellent signal for herbivores (Engelberth et al., 2004; Morrell & Kessler, 2017; Shannon et al., 2016). Receivers of herbivore‐induced signals can also produce secondary metabolites that limit the palatability of receiver plants when herbivores feed on them (Divekar et al., 2022; Himanen et al., 2010; Sugimoto et al., 2014). Both the nature and concentration of volatiles emitted after herbivore damage are highly variable within and among species (Chen et al., 2003; Degen et al., 2004; Dudareva et al., 2006; Kalske et al., 2019; Müller et al., 2020; Snoeren et al., 2010). Thus, plant–plant signalling can potentially evolve in few generations, depending on the herbivory pressure existing within populations (Kalske et al., 2019). Moreover, plant–plant signalling can be costly for plant fitness depending on the environmental conditions, and therefore be associated with adaptive trade‐offs. For instance, Benevenuto et al. (2020) recently showed that simulated attacks of herbivore on bilberry plants reduced leaf consumption on conspecific neighbours at low and mid altitude, but had no effect at high altitude, indicating potential costs of induced defences in stressful environments.

The highly cosmopolitan plant *Arabidopsis thaliana* (L.) is a powerful model to investigate intraspecific variability in plant–plant signalling and plant–herbivore interactions. *A. thaliana* is a small annual plant inhabiting contrasting environments and climates all over the world (1001 Genomes Consortium, 2016; Hoffmann, 2002; Lee et al., 2017). An international effort of sampling made available fully‐sequenced genomes for hundreds of natural genotypes (hereafter ‘accessions’) (1001 Genomes Consortium, 2016). This open‐access database is a great opportunity to study the genetic basis of phenotypic variation. For instance, genomic variation between accessions of *A. thaliana* showed evidence of local adaptation to climate (Clauw et al., 2022; Dittberner et al., 2018; Exposito‐Alonso, 2020; Lasky et al., 2014; Vasseur et al., 2018). Moreover, natural accessions of *A. thaliana* expressed contrasting values of constitutive defences against herbivores, notably in glucosinolate concentration in leaf tissues (Brachi et al., 2015; Gloss et al., 2017; Kerwin et al., 2015). Snoeren et al. (2010) showed that nine accessions of *A. thaliana* emitted different blends of volatile molecules in response to herbivore damage (see also Savchenko et al., 2013). Other studies showed that the Col‐0 accession increased gene expression associated with resistance functions after being exposed to particular volatile molecules such as aldehyde or monoterpenes (Kishimoto et al., 2005; Savchenko et al., 2013). Surprisingly though, the effect of a prior contact with herbivore‐induced signal on the foraging activity of an herbivore have been poorly investigated in this model species. The natural variation of the response of such herbivore‐induced volatiles within *A. thaliana* is also an opportunity to understand its genetic bases through genome‐wide association studies (GWAS). Such approaches are powerful tools to link complex phenotypes to genetic variation (Francisco et al., 2016).

**FIGURE 1:**
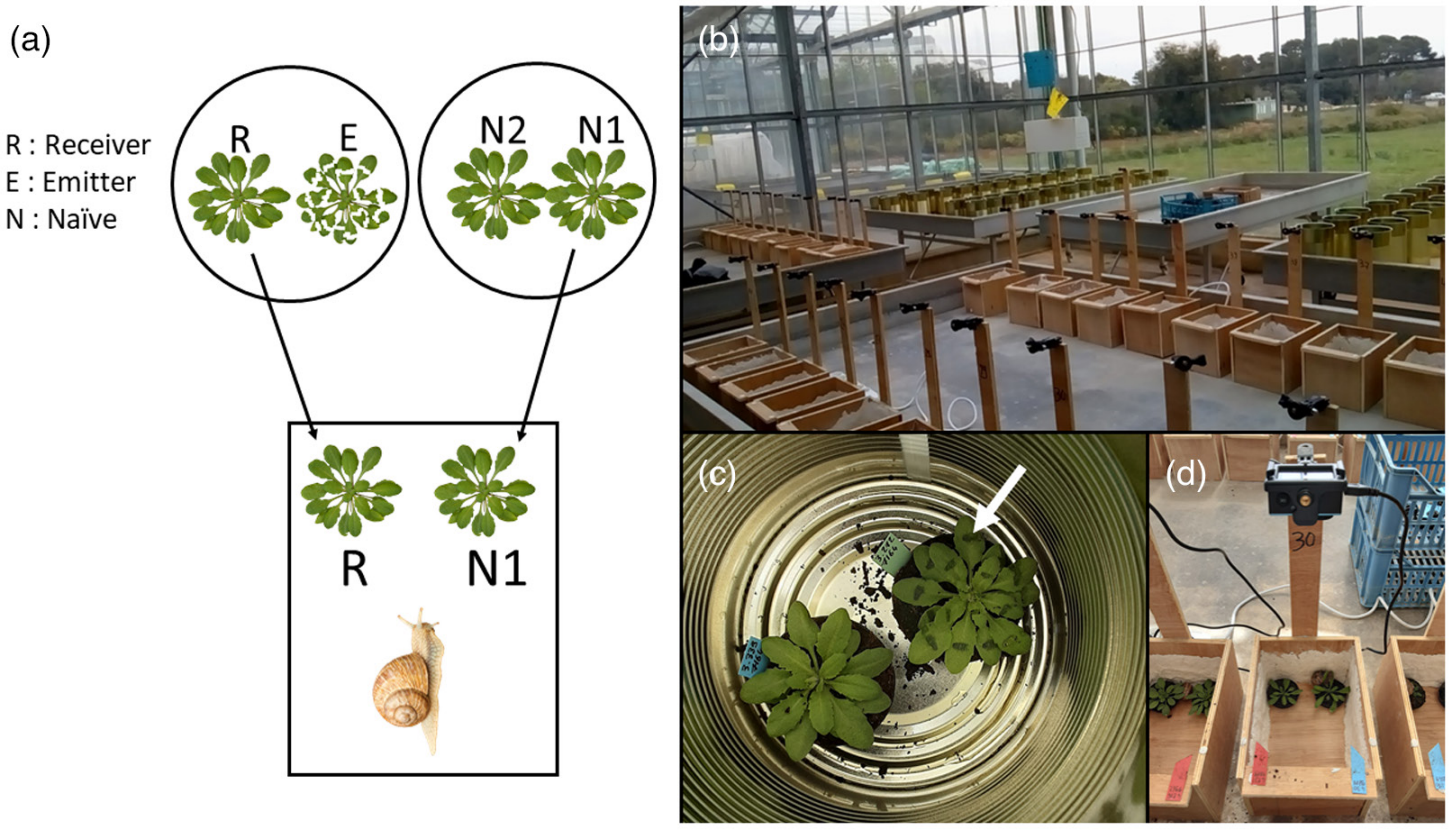
Experimental test of the effect of a prior contact with a damaged plant on the foraging activity of snails. (a) Scheme of the two stages of the experiment. (b) Picture of the greenhouse and of the experimental devices. (c) Details of a circular metallic enclosure. The contact between an emitter plant E (noted with an arrow, clipped) and a receiver plant R lasted 30 min in parallel of two intact plants (N1 and N2). (d) Wood arena where snail movements on R and N1 were filmed during 1 h.

Studies on plant–plant interactions in response to herbivory often used field‐based approaches (Benevenuto et al., 2020; Fowler & Lawton, 1985; Karban et al., 2000, 2003, 2016; Karban & Baxter, 2001; Morrell & Kessler, 2017; Pearse et al., 2013). Such an approach was associated with a dependence between the environment of the emitter and of the receiver. In other words, the signals emitted by the damaged plants may directly repel herbivores, providing indirect defences to neighbours (as discussed in Karban et al., 2014). Another aspect of field‐based approach is that leaf consumption on receiver plants of herbivore‐induced signals was mainly measured over long period of time (days, weeks or years) (Agrawal, 2000; Benevenuto et al., 2020; Fowler & Lawton, 1985; Karban et al., 2000, 2003, 2016; Morrell & Kessler, 2017; Pearse et al., 2013; Rhoades, 1983). How short‐term foraging activity of herbivores on plants is impacted when plants had a prior contact with a damaged neighbour may be crucial to understand plant–herbivore interaction mediated by plant–plant signalling (Morrell & Kessler, 2017). Here, we built an innovative experimental design (Figure 1) that allowed us to record the foraging activity of snails (*Cornu aspersum*, Müller, 1774) across pairs of plants differing only by a prior contact or not with an artificially damaged individual. We conducted a glasshouse experiment with 113 worldwide accessions of *A. thaliana* to investigate (i) whether the foraging activity of a generalist herbivore is impacted by the prior contact of a plant with another damaged plant? (ii) if plant–herbivore interaction mediated by a prior contact varies across natural accessions of *A. thaliana*? (iii) whether this potential variation is explained by allelic diversity between plant accessions?

## 2 MATERIALS AND METHODS

### 2.1 Plant material

We randomly selected 113 natural accessions of *A. thaliana* from the 1001 Genome germplasm and thus entirely sequenced (1001 Genomes Consortium, 2016). Seeds were obtained from NASC and were then reproduced in greenhouse (CEFE, Montpellier, France). We used garden snail (*C. aspersum*), a generalist herbivore, whose geographical range overlap the majority of the European distribution of *A. thaliana*. Snails used in this study were raised in an organic farm (L'escargot du Riberal, L. Sanchez, Tautavel, France). Snails were fed with industrial and specialized flour at the farm and were starved during 1 week in a cold room before the experiment.

### 2.2 Growth conditions

Seeds were sown at soil surface of 5‐cm pots (80 mL) previously filled with peat substrate (Neuhaus N2) and placed in greenhouse (Montpellier, France) in October 2020. We used eight consecutive sowings consisting of four seeds of the 113 accessions (*n* = 3616 pots in total), randomly arranged in eight blocks, one block corresponding to one sowing date. All blocks were rotated on a daily basis. All pots were sub‐irrigated with water treated by reverse osmosis to field‐capacity every 2–4 days. The temperature was set at 20°C during the day and 16°C during the night for the full duration of the experiment. Sciarid flies were observed 5 weeks after germination. We spread water‐diluted larvicide on the surface of all the pots (Vectobac WG, Edialux, France). Due to germination issues, 600 pots were removed. The 3016 remaining pots were undamaged by sciarid flies and represented between 20–32 individuals per accession.

### 2.3 Experimental design

Seven weeks after sowing, two pairs of individual plants of the same accession and from the same block were separately placed in circular metallic enclosures (diameter = 15 cm, height = 25 cm, Figure 1). In the first pair, all leaves of one individual (‘emitter plant’, E) were clipped with clamps in order to simulate herbivory (Figure 1a,c). The neighbour individual (‘receiver plant’, R) was kept intact. Both control individuals of the second pair were left undamaged (‘naive plants’ N1 and N2). The two pairs of neighbour plants remained 30 min in their respective enclosure after the treatment of emitter plant E. Then, the intact individuals from the two pairs (R and N1) were placed together in a wood arena (Figure 1d). A snail (*C. aspersum*) was released at equidistance of each plant and was filmed in the arena during 1 h (1 photo per second, 2.7 kppi, XPRO2, TecTecTec, France). An example of a video is available in the Video S1. We took pictures of the plants before and after the passage of the snail (16 Gpi, XPRO2, TecTecTec, France). We performed simultaneously 63 tests on a half day, one test corresponding to one replicate per accession. Positions of pairs of plants among the 126 metallic enclosures and among the 63 arenas were randomly assessed. All videos and images are available at https://doi.org/10.57745/QN7MMM.

### 2.4 Measurements

A binomial variable ‘First choice’ was scored 1 to every arena where the R plant was chosen first by the snail, and 0 elsewhere, i.e. to every arena where N1 plant was chosen first (Table 1). First duration was measured as the time spent by the snail on the first plant (Table 1). We measured the total duration that the snail spent on each individual plant during 1 h. The proportion of time was then calculated as the duration on one plant divided by the sum of the durations on the two plants in the arena (Table 1). We estimated a ‘leaf consumption index’ from the pictures taken after the passage of the snail (Table 1). We analysed individual pictures (and not pictures of pairs) in order to avoid biased results related to the leaf consumption index of the second individual of the pair. A value of zero was given if the snail did not consume any part of the plant and a maximum value of five if all the leaves of the plant were eaten by the snail (Figure S1). We confronted the leaf consumption index estimated by two independent observers on a training data set of 227 pictures to validate the method (*ρ* = 0.86, *p* < 0.0001). The initial rosette area of each plant was estimated from the picture taken before the passage of the snail (ImageJ; version 1.53c, Schneider et al., 2012). We then calculated the ‘rosette area difference’, as the difference between initial rosette areas of the R plant and the N1 plant in each arena.

**TABLE 1:** List of response variables and their observed range of variation.

	Unit	Range of variation	Related questions
First choice	Binomial	[0; 1]	Which plants were chosen first by the snail?
First duration	s	[16: 3700]	How much time did the snail spend on the first plant?
Proportion of time	%	[0: 100]	What is the proportion of time that the snail spent on R and N1 plants in an arena?
Leaf consumption index	–	[0; 1; 2; 3; 4; 5]	What quantity of leaves were consumed on R and on N1 plants within an arena?

### 2.5 Statistical analyses

All analyses were performed with R (version 4.0.5). We tested if a prior contact to a clipped plant and rosette area difference had an effect on each response variables separately. We used quasibinomial models to test these effects on the first choice, the proportion of time spent on R, and the proportion of leaf consumption index (link function ‘probit’). We used quasibinomial models instead of binomial model to correct for overdispersion. Quasibinomial models artificially increase standard errors, and are more conservative than binomial models (Dunn & Smyth, 2018). Effect on first duration was tested using a linear model, after log_10_‐transformation to fit normality assumptions. Intercepts and slopes estimated by models were used to quantify the effects of prior contact and total leaf area difference between plants, respectively. For example, for the variable ‘First choice’, a significant negative intercept indicates a weaker probability for R plants to be chosen first than N1 plants. A positive slope indicates a higher probability to choose R first than N1 if R plant was bigger than N1 plants.

The effect of accession identity was tested with linear model for the first duration and with quasibinomial models for the three other response variables. Rosette area difference was systematically added as a covariate in these models. The effect of accession identity was reported as its global effect on the variance (ANOVA of type II, package ‘car’). From these models, we calculated marginal means of the response variables of all accessions using the package ‘emmeans’ (Lenth et al., 2018). Correlations between mean responses of accessions were assessed with the Kendal's statistic.

### 2.6 Quantitative genetic analysis

We tested for allelic effects on the four response variables through genome‐wide association studies using the online platform EasyGWAS (https://easygwas.ethz.ch/, Grimm et al., 2017). Marginal means of accessions, as calculated above, were implemented. We used the EMMAX algorithm, filtering for minimum alleles frequency at 5% and including the two first axes of a PCA performed on SNPs to correct for population structure. Significance thresholds were assessed by the Bonferroni method with an alpha error of both 0.05 and 0.1. Known functions of genes were extracted from TAIR10 database (https://www.arabidopsis.org, Lamesch et al., 2012).

## 3 RESULTS

### 3.1 Prior exposure to a damaged neighbour and rosette area difference effects on snail foraging activity

**FIGURE 2:**
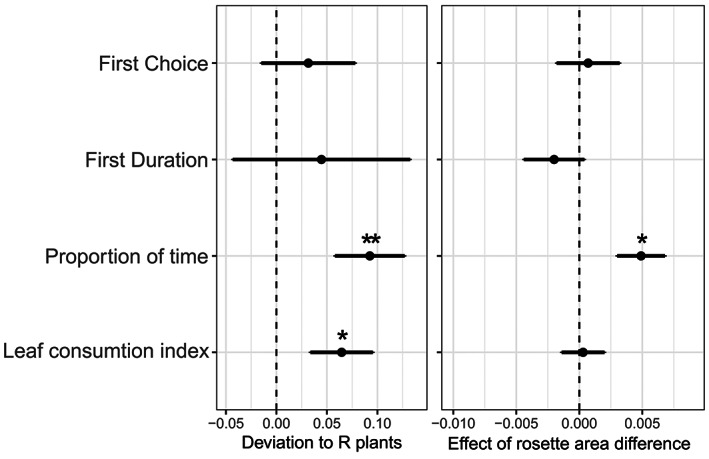
Prior contact and rosette area difference effects on snail foraging activity. Estimates and standard errors of the coefficients of models are represented here on a probit scale. *p < 0.05, **p < 0.01, ***p < 0.001.

Across all accessions, prior contact and rosette area difference did not have a significant effect on the first choice of the snail (*t* = 0.680, *p* = 0.50, and *t* = 0.266, *p =* 0.79, respectively, Figure 2) nor on the first duration spent on the first encountered individual (*t* = 0.508, *p* = 0.61 and *t* = −0.833, *p* = 0.41 respectively, Figure 2). The mean first duration on a plant was positively correlated with the mean rosette area (*ρ* = 0.3, *p* = 0.001).

Prior contact with a damaged neighbour (R plants) had a significant effect on the proportion of time spent on plants (*t* = 2.682, *p* < 0.001). Over all accessions, snails spent proportionally more time on R plants (54 ± 1.9%), i.e. plants previously exposed to artificially damaged plants, than on control N1 plants (46 ± 1.35%), i.e. plants that were previously adjacent to undamaged plants (Figure 2). In addition, difference in rosette area between the two plants in the arena had a significant and positive effect on the proportion of time spent on R plants (*t* = 2.627, *p* < 0.001, Figure 2). In other words, snails spent more time on the R plant if the R plant had a bigger rosette than N1 plant, in addition to the effect of a prior contact.

A prior exposure to a damaged neighbour plant had a significant effect on leaf consumption index. Leaf consumption index was higher for R plants than for N1 plants (*t* = 2.089, *p* < 0.05, Figure 2). Leaf consumption index was highly correlated with the proportion of time spent on plants (*ρ* = 0.46, *p* < 0.001), but not significantly explained by rosette area difference (*t* = 0.153, *p* = 0.88, Figure 2).

### 3.2 Genotypic and allelic effects of attractivity of plants exposed to a damaged neighbour

**FIGURE 3:**
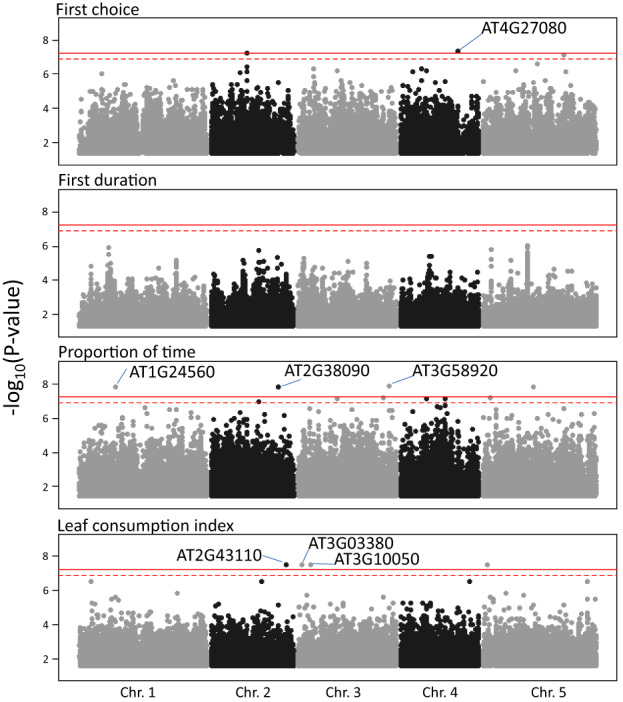
Genome‐wide association of the four response variables. GWAS were performed with EMMAX across 113 natural accessions on 1,922,479 SNPs. Each dot is a SNP located on chromosomes 1, 3 and 5 (grey) and chromosomes 2 and 4 (black). Bonferroni threshold at 0.05 and 0.1 was represented with red solid and dashed lines, respectively.

The probability to choose R plants first varied a lot between plant accessions, ranging from 12.5% (1/8) to 100% (8/8) across accessions. Two SNPs were associated with the first choice of snails on R plants (Figure 3). No gene was found at the first SNP on the second chromosome, but this SNP was upstream to AT2G19800, a gene that encodes a myo‐inositol oxygenase (MIOX). MIOX gene family is involved in oxidative stress tolerance (Munir et al., 2020). On the fourth chromosome, a significant SNP was located in AT4G27080, a gene that encodes a disulphide isomerase‐like (PDIL) protein (Table S1), involved in biotic and abiotic stress tolerance (Chen et al., 2012; Wang et al., 2019).

The ‘first duration’ variable exhibited a bimodal distribution, reflecting that, depending on the accession, the time spent by a snail on the first plant chosen was brief (16.2–500 s) or long (more than 2000 s), with fewer intermediate durations (Figure S2). The identity of the accession explained a significant part of the variance of the first duration spent on a plant (20% of the total variance, *p* = 0.02). However, no SNP position along the genome was significantly associated with the first duration spent on a plant (Figure 3, Table S2).

We found a large variation in the proportion of time spent on R plants across accessions (16%–100%). Four SNPs along the genomes were significantly associated with the proportion of time spent on R plants (Figure 3). Among them, the gene AT1G24560.1 encodes for plant‐unique RAB5 effector 2 (PUF2), a key protein in cell‐to‐cell communication in plants (Table S3).

The leaf consumption index on R plants relative to N1 plants was significantly explained by accession identity (*χ* ^2^ = 164, *df* = 112, *p* < 0.001). In other words, depending on accession identity, a prior exposure to a damaged plant can increase or decrease the attractiveness of undamaged plants. Along the genome, four SNPs were significantly associated to the proportion of leaf consumption index on R plants (Figure 3). Among them, AT3G10050.1 encodes for the first enzyme in the biosynthetic pathway of isoleucine. Another gene was found on chromosome 3 (AT3G03380.1) that encodes for a periplasmic protease (Table S4), a protein involved in stress tolerance (Luciński et al., 2011). The effects and the frequencies of minor alleles for every significant SNP are detailed in Table S5.

## 4 DISCUSSION

For the last decades, consistent progress has been made in the characterization of plant–plant signalling and its effect on herbivore foraging activity (Agrawal, 2000; Dicke & Baldwin, 2010; Heil & Karban, 2010; Karban, 2021). Within‐species variation has been however overlooked, despite its central place in local adaptation. Here, we showed that a prior contact with a damaged plant had, on average, a negative effect on *A. thaliana*'s defence against a generalist herbivore, as snails spent significantly more time and ate more on receiver plants. We also found that this response is highly variable between accessions, and that allelic diversity underlies this variation.

A contact with a damaged plant led to higher attractivity of *A. thaliana* for snails, in terms of time spent on plants and of leaf consumption. Our results confirmed that plant–herbivore interactions can be partly regulated by plant–plant signalling (Dicke & Baldwin, 2010; Karban et al., 2014). However, our results contradict the general idea that plant–plant signalling has been selected to promote deterrence to herbivore attack (see Karban et al., 2014; Karban, 2021; Pearse & Karban, 2013 for reviews). Snails may be attracted to plants previously exposed to damaged neighbours for many reasons as developed in the following paragraphs.

First, odour and taste of the emitter damaged plant can have been deposited on the neighbouring plants (Matsui, 2016). Snails possess the ability to sense odour and to discriminate them in a saturated context (Shannon et al., 2016). In the arena, the plant previously exposed to a damaged neighbour, i.e., receiver plant, can thus exhibit a different olfactory signal compared with the plant exposed to an undamaged neighbour. Indeed, snails significantly preferred damaged plants compared with undamaged plants (Appendix S1). Attractiveness of damaged plants has been shown in numerous species of insect herbivores (Bolter et al., 1997; Carroll et al., 2006; Harari et al., 1994; Loughrin et al., 1995). Life‐style of herbivores, such as aggregating behaviour are aspects that are likely to be part of the attractiveness of damaged plants and of their neighbours (Bolter et al., 1997). The search for mating opportunities may explain such attraction for damaged plants by conspecifics (Arab et al., 2007; Kalberer et al., 2001). In case of uncertainty of feeding, a choice on a sub‐optimal but detectable plant may also be the better choice for a slow‐foraging herbivore (Carroll et al., 2006).

Second, interactions between two individuals of *A. thaliana* can also be maladaptive or addressed to species other than snails. To our knowledge, only one case of increased attraction to herbivores in response to plant–plant signalling has been reported (Zhang et al., 2019). In this example, tomato fruits infested by the whitefly *Bemisia tabacito* expressed a defence against pathogens instead of herbivores. Neighbours of such manipulated plants also expressed unappropriated defences and thus were more attractive to whiteflies (Zhang et al., 2019). In addition, recent studies on *Baccharis salicifolia* showed that the volatiles emitted consecutively to herbivore damages, and their effects on neighbours was highly specialized to herbivore species (Moreira et al., 2018; and see Moreira & Abdala‐Roberts, 2019 for a review). As we simulated herbivore attack by clipping the leaves of *A. thaliana*, induced defences may not be specifically addressed to snails. Numerous studies showed that herbivore‐induced volatiles attract parasitoid insects in plant species, including *A. thaliana* that represent indirect defences to herbivores (Baldwin et al., 2002; Dicke & Baldwin, 2010; Girling et al., 2006; Kessler & Baldwin, 2001; Turlings et al., 1990; Turlings & Erb, 2018; Van Poecke et al., 2001). Such indirect defence through parasitoid attraction may be involved in our study but would require additional manipulations.

Third, the biased foraging activity of snails on plants that experienced a previous contact with a damaged plant was accentuated for accessions that originated from high latitudes and from environment with low mean annual temperatures (Figure S3). Long‐standing and highly‐debated hypotheses on latitudinal gradient in herbivory pressure may be associated to such latitudinally structured genotypic variability (Anstett et al., 2016). Our results echoed the study of Benevenuto et al. (2020) that showed that plant–plant signalling effect on plant defence induction against herbivores decreased with altitude in wild populations of bilberries (*Vaccinium myrtillus* L.). Costs involved in the induction of defences against herbivores by plant–plant signalling may overtake benefits for individual from harsh, stressful environments (Strauss et al., 2002). Such results highlight the necessity of studies of intraspecific diversity along environmental gradients of plant–plant signalling and plant–herbivore interactions to better understand their evolution.

A key aspect of modern biology is to understand how complex phenotypes are genetically controlled (Francisco et al., 2016). Genome‐wide association studies are powerful and fast tools for gene hunting (Brachi et al., 2015; Francisco et al., 2016; Gloss et al., 2017). The completely‐sequenced genomes of thousands of natural accessions of *A. thaliana* is then the best material for such genotype‐to‐phenotype studies (1001 Genomes Consortium, 2016). Here, we found that the effect of previous plant exposure to a damaged neighbour on snail behaviour relied on several SNPs along the genome. Most of them were associated with regions with unknown functions, thus opening the way to the exploration of new pathways involved in plant defences (Tables [Link], [Link]). Other significant SNPs were related to plant ability to cope with stress. For instance, AT2G43110 is involved in the regulation of drought tolerance (Noman et al., 2019). AT3G03380 increases stress protection of the photosystem II in *A. thaliana* (Luciński et al., 2011). Interestingly, we found that variation in leaf consumption was associated with the AT3G10050 gene, which encodes the first enzyme in the synthetic pathway of isoleucine, an activator of jasmonic acid (Staswick & Tiryaki, 2004) that is a well‐known molecule involved in plant–plant and plant–herbivore interactions (Ray et al., 2019). Although these genes remain to be validated, for instance using mutant phenotype expressions, they are promising candidates to better understand the mechanisms involved in plant defences against herbivore mediated by plant–plant interactions. If validated, such genetic variation underlying plant–plant interactions for induced defence against herbivore may rise opportunities for plant breeding in an agricultural context. For example, the push‐pull strategy used in hundreds of sub‐Saharan fields, consisting in mixing attractive and repulsive species within a field, may enhanced both ‘pull’ and ‘push’ compartments with artificial selection on alleles of interest (Bruce, 2010; Guerrieri, 2016; Pickett & Khan, 2016; Turlings & Erb, 2018). A breeding for such plant–plant interactions within species may also enhance yield stability in varietal mixtures (Pélissier et al., 2021).

## 5 CONCLUSION

How plant–plant interactions could evolve in response to herbivory is highly debated (Dicke & Baldwin, 2010; Heil, 2014; Heil & Adame‐Álvarez, 2010; Heil & Karban, 2010; Karban, 2021; Karban et al., 2014). Discussions were substantial on the advantage for an attacked plant to produce a signal that warns and helps potential competitors (Agrawal, 2000; Heil & Adame‐Álvarez, 2010; Heil & Karban, 2010). At the opposite, the advantages for a plant receptor of such signal were often considered as adaptive, in the case of increased resistance to herbivory after being in contact with a damaged plant. Theoretical expectations on the evolution of an increased attractivity to herbivores when receiving cues from attacked plants are missing in the literature (as discussed in Pearse & Karban, 2013). Promoting continuous movement of herbivores between attractive plants may limit biomass consumption at the group level (Morrell & Kessler, 2017). The important genotypic variation in plant–plant signalling and its effect on plant–herbivore interactions found in our study raises important questions regarding its adaptive significance. Our results open avenues for designing more complex studies, notably to test the effect of genetical or morphological distances between plants, to disentangle the role of plant–plant signalling on plant–herbivore interactions.

## CONFLICT OF INTEREST STATEMENT

The authors have no conflict of interest to declare.

## Supporting information


Figure S1.

Figure S2.

Figure S3.

Video S1.

Table S1.

Table S2.

Table S3.

Table S4.

Table S5.

Appendix S1.

## Data Availability

The data that support the findings of this study are openly available in Data.INRAE at https://doi.org/10.57745/QN7MMM.
