# Supplementary figures and images for: Plant–herbivore interactions: Experimental demonstration of genetic variability in plant–plant signalling
Source: Evol Appl. 2023 Mar 29;16(4):772–80. doi: 10.1111/eva.13531 (PMC10130558; doi:10.1111/eva.13531)

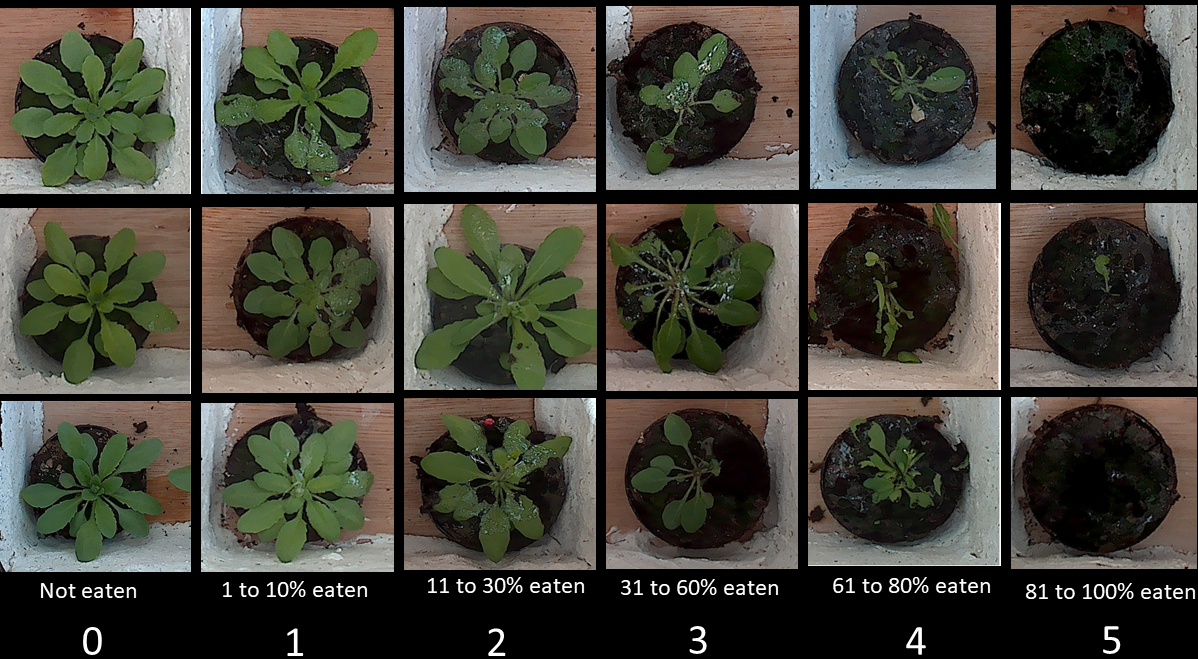

Supplement: Supplementary file 1 — Figure S1. [file EVA-16-772-s006.png]

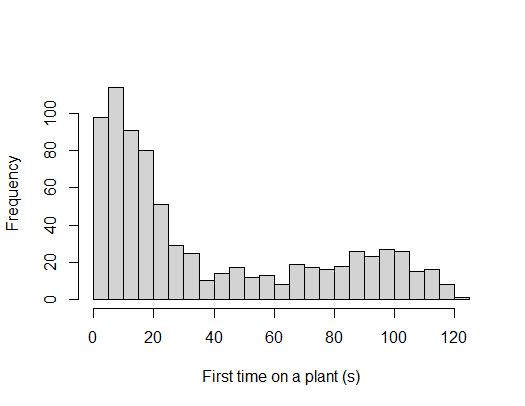

Supplement: Supplementary file 2 — Figure S2. [file EVA-16-772-s004.png]
