# Supplementary material for: Plant–herbivore interactions: Experimental demonstration of genetic variability in plant–plant signalling
Source: Evol Appl. 2023 Mar 29;16(4):772–80. doi: 10.1111/eva.13531 (PMC10130558; doi:10.1111/eva.13531)
Supplement: Supplementary file 3 — Figure S3. [file EVA-16-772-s010.docx]

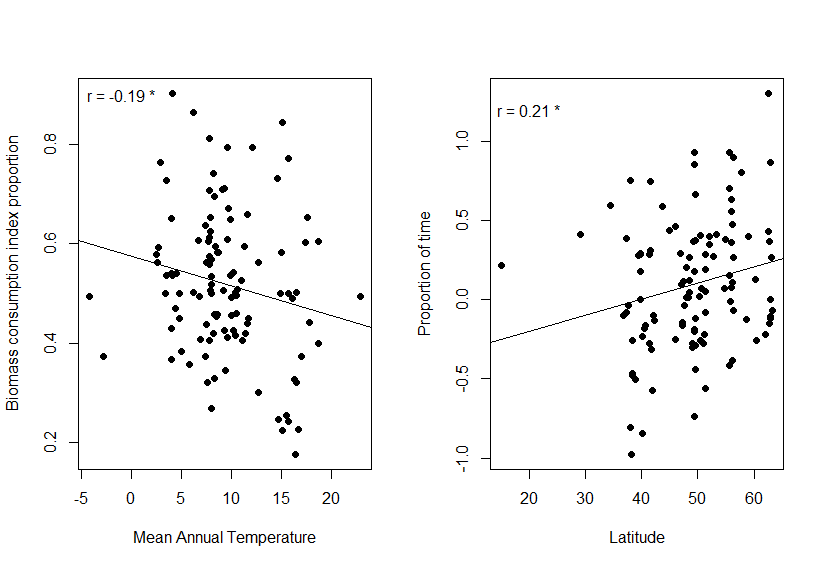


**Figure S3.** Correlation of plant-plant signaling effect on plant-herbivore interaction with temperature and latitudes. The other response variables were uncorrelated with climatic variables, latitudes nor longitudes (not shown). Statistics presented here are coefficient of correlation of Pearson. Such results may indicate that climatic conditions constrained the evolution of plant defense against herbivores through plant-plant signaling. Specific test has to be conducted, for instance *in natura*.
