# Supplementary material for: Plant–herbivore interactions: Experimental demonstration of genetic variability in plant–plant signalling
Source: Evol Appl. 2023 Mar 29;16(4):772–80. doi: 10.1111/eva.13531 (PMC10130558; doi:10.1111/eva.13531)
Supplement: Supplementary file 5 — Table S1. [file EVA-16-772-s007.docx]

**Table S1.** List of single nucleotid polymorphisms associated with the first choice. The 10 most associated SNPs are presented for each chromosome. P-values corresponded to SNPs association tests with the response variable. Significance threshold indicate if the p-values of SNP outreached Bonferonni correction at 0.05 or 0.1 threshold.

| **Chromosome** | **Position** | **P-Value** | **Gene** | **Significance threshold** |
| --- | --- | --- | --- | --- |
| Chr1 | 5406718 | 9.484e-07 | [AT1G15720](http://arabidopsis.org/servlets/TairObject?name=AT1G15720.1&type=gene) |  |
| Chr1 | 22372448 | 2.510e-06 | No Gene found |  |
| Chr1 | 10870243 | 3.889e-06 | [AT1G30650](http://arabidopsis.org/servlets/TairObject?name=AT1G30650.1&type=gene) |  |
| Chr1 | 10869720 | 3.889e-06 | [AT1G30650](http://arabidopsis.org/servlets/TairObject?name=AT1G30650.1&type=gene) |  |
| Chr1 | 10772586 | 3.889e-06 | [AT1G30460](http://arabidopsis.org/servlets/TairObject?name=AT1G30460.1&type=gene) |  |
| Chr1 | 10743223 | 3.889e-06 | [AT1G30410](http://arabidopsis.org/servlets/TairObject?name=AT1G30410.1&type=gene) |  |
| Chr1 | 10814390 | 3.889e-06 | No Gene found |  |
| Chr1 | 10857264 | 3.889e-06 | [AT1G30620](http://arabidopsis.org/servlets/TairObject?name=AT1G30620.1&type=gene) |  |
| Chr1 | 10867079 | 3.889e-06 | No Gene found |  |
| Chr1 | 10778403 | 3.889e-06 | No Gene found |  |
| **Chr2** | **8535984** | **5.523e-08** | **No Gene found** | **< 0.05** |
| Chr2 | 8532837 | 3.530e-07 | [AT2G19800](http://arabidopsis.org/servlets/TairObject?name=AT2G19800.1&type=gene) |  |
| Chr2 | 8533232 | 3.530e-07 | [AT2G19800](http://arabidopsis.org/servlets/TairObject?name=AT2G19800.1&type=gene) |  |
| Chr2 | 8532810 | 3.530e-07 | [AT2G19800](http://arabidopsis.org/servlets/TairObject?name=AT2G19800.1&type=gene) |  |
| Chr2 | 8533424 | 3.530e-07 | [AT2G19800](http://arabidopsis.org/servlets/TairObject?name=AT2G19800.1&type=gene) |  |
| Chr2 | 8532824 | 3.530e-07 | [AT2G19800](http://arabidopsis.org/servlets/TairObject?name=AT2G19800.1&type=gene) |  |
| Chr2 | 8532794 | 3.530e-07 | [AT2G19800](http://arabidopsis.org/servlets/TairObject?name=AT2G19800.1&type=gene) |  |
| Chr2 | 8533201 | 3.530e-07 | [AT2G19800](http://arabidopsis.org/servlets/TairObject?name=AT2G19800.1&type=gene) |  |
| Chr2 | 8532826 | 3.530e-07 | [AT2G19800](http://arabidopsis.org/servlets/TairObject?name=AT2G19800.1&type=gene) |  |
| Chr2 | 8533191 | 3.530e-07 | [AT2G19800](http://arabidopsis.org/servlets/TairObject?name=AT2G19800.1&type=gene) |  |
| Chr3 | 3736405 | 4.693e-07 | [AT3G11840](http://arabidopsis.org/servlets/TairObject?name=AT3G11840.1&type=gene) |  |
| Chr3 | 9387772 | 6.758e-07 | No Gene found |  |
| Chr3 | 3755234 | 1.439e-06 | No Gene found |  |
| Chr3 | 10141107 | 2.354e-06 | [AT3G27400](http://arabidopsis.org/servlets/TairObject?name=AT3G27400.1&type=gene) |  |
| Chr3 | 12423592 | 3.038e-06 | No Gene found |  |
| Chr3 | 3880089 | 3.284e-06 | [AT3G12160](http://arabidopsis.org/servlets/TairObject?name=AT3G12160.1&type=gene) |  |
| Chr3 | 3872474 | 4.056e-06 | No Gene found |  |
| Chr3 | 3874836 | 4.056e-06 | [AT3G12145](http://arabidopsis.org/servlets/TairObject?name=AT3G12145.1&type=gene) |  |
| Chr3 | 3873803 | 4.056e-06 | No Gene found |  |
| Chr3 | 10535319 | 4.329e-06 | [AT3G28260](http://arabidopsis.org/servlets/TairObject?name=AT3G28260.1&type=gene) |  |
| **Chr4** | **13591215** | **4.250e-08** | [**AT4G27080**](http://arabidopsis.org/servlets/TairObject?name=AT4G27080.1&type=gene) | **< 0.05** |
| Chr4 | 4963674 | 4.964e-07 | No Gene found |  |
| Chr4 | 6192309 | 6.758e-07 | No Gene found |  |
| Chr4 | 2770763 | 7.470e-07 | No Gene found |  |
| Chr4 | 4963597 | 2.389e-06 | No Gene found |  |
| Chr4 | 10513229 | 2.859e-06 | No Gene found |  |
| Chr4 | 8128857 | 3.183e-06 | [AT4G14104](http://arabidopsis.org/servlets/TairObject?name=AT4G14104.1&type=gene) |  |
| Chr4 | 8128184 | 3.183e-06 | [AT4G14103](http://arabidopsis.org/servlets/TairObject?name=AT4G14103.1&type=gene) |  |
| Chr4 | 553035 | 4.912e-06 | No Gene found |  |
| Chr4 | 1559940 | 5.601e-06 | No Gene found |  |
| **Chr5** | **19126928** | **7.937e-08** | **No Gene found** | **< 0.1** |
| Chr5 | 12936199 | 2.665e-07 | No Gene found |  |
| Chr5 | 12936178 | 2.665e-07 | No Gene found |  |
| Chr5 | 7814665 | 6.606e-07 | [AT5G23210](http://arabidopsis.org/servlets/TairObject?name=AT5G23210.1&type=gene) |  |
| Chr5 | 19676706 | 7.119e-07 | [AT5G48545](http://arabidopsis.org/servlets/TairObject?name=AT5G48545.1&type=gene) |  |
| Chr5 | 14898461 | 2.385e-06 | [AT5G37510](http://arabidopsis.org/servlets/TairObject?name=AT5G37510.1&type=gene) |  |
| Chr5 | 14895847 | 2.385e-06 | No Gene found |  |
| Chr5 | 156685 | 2.632e-06 | [AT5G01380](http://arabidopsis.org/servlets/TairObject?name=AT5G01380.1&type=gene) |  |
| Chr5 | 156533 | 2.632e-06 | [AT5G01380](http://arabidopsis.org/servlets/TairObject?name=AT5G01380.1&type=gene) |  |
| Chr5 | 156561 | 2.632e-06 | [AT5G01380](http://arabidopsis.org/servlets/TairObject?name=AT5G01380.1&type=gene) |  |
