# Supplementary material for: Plant–herbivore interactions: Experimental demonstration of genetic variability in plant–plant signalling
Source: Evol Appl. 2023 Mar 29;16(4):772–80. doi: 10.1111/eva.13531 (PMC10130558; doi:10.1111/eva.13531)
Supplement: Supplementary file 6 — Table S2. [file EVA-16-772-s009.docx]

**Table S2.** List of single nucleotid polymorphisms associated with the first duration. The 10 most associated SNPs are presented for each chromosome. P-values corresponded to SNPs association tests with the response variable. Significance threshold indicate if the p-values of SNP outreached Bonferonni correction at 0.05 or 0.1 threshold.

| **Chromosome** | **Position** | **P-Value** | **Gene** | **Significance threshold** |
| --- | --- | --- | --- | --- |
| Chr1 | 6868887 | 1.159e-06 | No Gene found |  |
| Chr1 | 6869063 | 3.029e-06 | No Gene found |  |
| Chr1 | 23193564 | 6.827e-06 | [AT1G62640](http://arabidopsis.org/servlets/TairObject?name=AT1G62640.1&type=gene) |  |
| Chr1 | 23194596 | 6.827e-06 | [AT1G62640](http://arabidopsis.org/servlets/TairObject?name=AT1G62640.1&type=gene) |  |
| Chr1 | 23193553 | 6.827e-06 | [AT1G62640](http://arabidopsis.org/servlets/TairObject?name=AT1G62640.1&type=gene) |  |
| Chr1 | 23193539 | 6.827e-06 | [AT1G62640](http://arabidopsis.org/servlets/TairObject?name=AT1G62640.1&type=gene) |  |
| Chr1 | 7464568 | 1.009e-05 | [AT1G21320](http://arabidopsis.org/servlets/TairObject?name=AT1G21320.1&type=gene) |  |
| Chr1 | 23194570 | 1.034e-05 | [AT1G62640](http://arabidopsis.org/servlets/TairObject?name=AT1G62640.1&type=gene) |  |
| Chr1 | 23180501 | 1.244e-05 | [AT1G62600](http://arabidopsis.org/servlets/TairObject?name=AT1G62600.1&type=gene) |  |
| Chr1 | 6737352 | 1.345e-05 | No Gene found |  |
| Chr2 | 11402355 | 1.690e-06 | [AT2G26760](http://arabidopsis.org/servlets/TairObject?name=AT2G26760.1&type=gene) |  |
| Chr2 | 15631446 | 4.650e-06 | No Gene found |  |
| Chr2 | 15659311 | 4.650e-06 | [AT2G37290](http://arabidopsis.org/servlets/TairObject?name=AT2G37290.1&type=gene) |  |
| Chr2 | 15659845 | 4.650e-06 | [AT2G37290](http://arabidopsis.org/servlets/TairObject?name=AT2G37290.1&type=gene) |  |
| Chr2 | 7529492 | 7.091e-06 | No Gene found |  |
| Chr2 | 12361132 | 7.842e-06 | No Gene found |  |
| Chr2 | 12361120 | 7.842e-06 | No Gene found |  |
| Chr2 | 12361081 | 7.842e-06 | No Gene found |  |
| Chr2 | 11402380 | 8.871e-06 | [AT2G26760](http://arabidopsis.org/servlets/TairObject?name=AT2G26760.1&type=gene) |  |
| Chr2 | 11415746 | 8.871e-06 | [AT2G26780](http://arabidopsis.org/servlets/TairObject?name=AT2G26780.1&type=gene) |  |
| Chr3 | 1342272 | 5.407e-06 | No Gene found |  |
| Chr3 | 11899159 | 7.509e-06 | No Gene found |  |
| Chr3 | 11898909 | 7.509e-06 | No Gene found |  |
| Chr3 | 1343283 | 9.232e-06 | [AT3G04870](http://arabidopsis.org/servlets/TairObject?name=AT3G04870.1&type=gene) |  |
| Chr3 | 1143595 | 9.796e-06 | No Gene found |  |
| Chr3 | 16282682 | 9.814e-06 | No Gene found |  |
| Chr3 | 16282723 | 9.814e-06 | No Gene found |  |
| Chr3 | 1342101 | 1.205e-05 | No Gene found |  |
| Chr3 | 1342122 | 1.205e-05 | No Gene found |  |
| Chr3 | 16283295 | 1.596e-05 |  |  |
| Chr4 | 6803323 | 3.748e-06 | No Gene found |  |
| Chr4 | 7281587 | 3.894e-06 | [AT4G12210](http://arabidopsis.org/servlets/TairObject?name=AT4G12210.1&type=gene) |  |
| Chr4 | 6791018 | 1.263e-05 | No Gene found |  |
| Chr4 | 6881035 | 2.431e-05 | No Gene found |  |
| Chr4 | 11742864 | 3.195e-05 | [AT4G22190](http://arabidopsis.org/servlets/TairObject?name=AT4G22190.1&type=gene) |  |
| Chr4 | 18320362 | 3.498e-05 | No Gene found |  |
| Chr4 | 18319793 | 3.498e-05 | No Gene found |  |
| Chr4 | 6482794 | 3.758e-05 | No Gene found |  |
| Chr4 | 7272980 | 3.775e-05 | No Gene found |  |
| Chr4 | 7279759 | 5.090e-05 | No Gene found |  |
| Chr5 | 10610684 | 9.629e-07 | No Gene found |  |
| Chr5 | 10609042 | 1.176e-06 | No Gene found |  |
| Chr5 | 1990083 | 1.564e-06 | [AT5G06530](http://arabidopsis.org/servlets/TairObject?name=AT5G06530.1&type=gene) |  |
| Chr5 | 10574472 | 2.263e-06 | No Gene found |  |
| Chr5 | 10549870 | 2.599e-06 | No Gene found |  |
| Chr5 | 10611001 | 2.758e-06 | [AT5G28610](http://arabidopsis.org/servlets/TairObject?name=AT5G28610.1&type=gene) |  |
| Chr5 | 10554396 | 3.681e-06 | No Gene found |  |
| Chr5 | 10546524 | 3.795e-06 | No Gene found |  |
| Chr5 | 10605404 | 3.853e-06 | No Gene found |  |
| Chr5 | 10605291 | 3.853e-06 | No Gene found |  |
