# Supplementary material for: Plant–herbivore interactions: Experimental demonstration of genetic variability in plant–plant signalling
Source: Evol Appl. 2023 Mar 29;16(4):772–80. doi: 10.1111/eva.13531 (PMC10130558; doi:10.1111/eva.13531)
Supplement: Supplementary file 7 — Table S3. [file EVA-16-772-s001.docx]

**Table S3.** List of single nucleotid polymorphisms associated with the proportion of time. The 10 most associated SNPs are presented for each chromosome. P-values corresponded to SNPs association tests with the response variable. Significance threshold indicate if the p-values of SNP outreached Bonferonni correction at 0.05 or 0.1 threshold.

| **Chromosome** | **Position** | **P-Value** | **Gene** | **Significance threshold** |
| --- | --- | --- | --- | --- |
| Chr1 | 8702745 | 1.447e-08 | [AT1G24560](http://arabidopsis.org/servlets/TairObject?name=AT1G24560.1&type=gene) | **< 0.05** |
| Chr1 | 15661471 | 2.492e-07 | No Gene found |  |
| Chr1 | 21410947 | 2.940e-07 | [AT1G57800](http://arabidopsis.org/servlets/TairObject?name=AT1G57800.1&type=gene) |  |
| Chr1 | 22977796 | 3.172e-07 | [AT1G62181](http://arabidopsis.org/servlets/TairObject?name=AT1G62181.1&type=gene) |  |
| Chr1 | 26470314 | 4.820e-07 | No Gene found |  |
| Chr1 | 16074152 | 5.149e-07 | No Gene found |  |
| Chr1 | 10146995 | 8.505e-07 | No Gene found |  |
| Chr1 | 26528424 | 8.915e-07 | No Gene found |  |
| Chr1 | 26528415 | 8.915e-07 | No Gene found |  |
| Chr1 | 28312332 | 8.947e-07 | No Gene found |  |
| Chr2 | 15945466 | 1.361e-08 | [AT2G38090](http://arabidopsis.org/servlets/TairObject?name=AT2G38090.1&type=gene) | **< 0.05** |
| Chr2 | 11284369 | 1.062e-07 | No Gene found | **< 0.1** |
| Chr2 | 6899677 | 4.630e-07 | No Gene found |  |
| Chr2 | 6899569 | 4.630e-07 | No Gene found |  |
| Chr2 | 12662006 | 6.292e-07 | No Gene found |  |
| Chr2 | 12661991 | 6.292e-07 | No Gene found |  |
| Chr2 | 12662000 | 6.292e-07 | No Gene found |  |
| Chr2 | 12662014 | 6.292e-07 | No Gene found |  |
| Chr2 | 17443744 | 7.064e-07 | No Gene found |  |
| Chr2 | 17443724 | 7.064e-07 | No Gene found |  |
| Chr3 | 21777184 | 1.350e-08 | [AT3G58920](http://arabidopsis.org/servlets/TairObject?name=AT3G58920.1&type=gene) | **< 0.1** |
| Chr3 | 21777182 | 1.350e-08 | [AT3G58920](http://arabidopsis.org/servlets/TairObject?name=AT3G58920.1&type=gene) | **< 0.1** |
| Chr3 | 20353154 | 6.404e-08 | No Gene found | **< 0.1** |
| Chr3 | 9387772 | 7.129e-08 | No Gene found | **< 0.1** |
| Chr3 | 2763336 | 2.644e-07 | [AT3G09040](http://arabidopsis.org/servlets/TairObject?name=AT3G09040.1&type=gene) |  |
| Chr3 | 2760726 | 2.644e-07 | No Gene found |  |
| Chr3 | 12690308 | 3.231e-07 | No Gene found |  |
| Chr3 | 12692133 | 3.231e-07 | No Gene found |  |
| Chr3 | 12693977 | 3.231e-07 | No Gene found |  |
| Chr3 | 12692027 | 3.231e-07 | No Gene found |  |
| Chr4 | 6192309 | 7.129e-08 | No Gene found | **< 0.1** |
| Chr4 | 10458151 | 7.355e-08 | [AT4G19110](http://arabidopsis.org/servlets/TairObject?name=AT4G19110.1&type=gene) | **< 0.1** |
| Chr4 |  |  | [AT4G19112](http://arabidopsis.org/servlets/TairObject?name=AT4G19112.1&type=gene) | **< 0.1** |
| Chr4 | 10458152 | 7.355e-08 | [AT4G19110](http://arabidopsis.org/servlets/TairObject?name=AT4G19110.1&type=gene) | **< 0.1** |
| Chr4 |  |  | [AT4G19112](http://arabidopsis.org/servlets/TairObject?name=AT4G19112.1&type=gene) | **< 0.1** |
| Chr4 | 10457385 | 7.355e-08 | [AT4G19110](http://arabidopsis.org/servlets/TairObject?name=AT4G19110.1&type=gene) | **< 0.1** |
| Chr4 |  |  | [AT4G19112](http://arabidopsis.org/servlets/TairObject?name=AT4G19112.1&type=gene) | **< 0.1** |
| Chr4 | 10434879 | 1.800e-07 | [AT4G19040](http://arabidopsis.org/servlets/TairObject?name=AT4G19040.1&type=gene) |  |
| Chr4 | 8768039 | 1.937e-07 | No Gene found |  |
| Chr4 | 9393772 | 2.469e-07 | No Gene found |  |
| Chr4 | 2770763 | 4.161e-07 | No Gene found |  |
| Chr4 | 10972352 | 5.382e-07 | No Gene found |  |
| Chr4 | 8350430 | 1.123e-06 | No Gene found |  |
| Chr5 | 12097495 | 1.361e-08 | No Gene found | **< 0.05** |
| Chr5 | 12097363 | 1.361e-08 | No Gene found | **< 0.05** |
| Chr5 | 1608450 | 6.404e-08 | No Gene found | **< 0.1** |
| Chr5 | 19126928 | 2.509e-07 | No Gene found |  |
| Chr5 | 3080093 | 3.259e-07 | [AT5G09876](http://arabidopsis.org/servlets/TairObject?name=AT5G09876.1&type=gene) |  |
| Chr5 | 26502544 | 5.460e-07 | [AT5G66340](http://arabidopsis.org/servlets/TairObject?name=AT5G66340.1&type=gene) |  |
| Chr5 | 15581992 | 5.788e-07 | [AT5G38920](http://arabidopsis.org/servlets/TairObject?name=AT5G38920.1&type=gene) |  |
| Chr5 | 3055123 | 6.159e-07 | No Gene found |  |
| Chr5 | 23475246 | 6.213e-07 | No Gene found |  |
| Chr5 | 6894748 | 6.934e-07 | No Gene found |  |
