# Supplementary material for: Plant–herbivore interactions: Experimental demonstration of genetic variability in plant–plant signalling
Source: Evol Appl. 2023 Mar 29;16(4):772–80. doi: 10.1111/eva.13531 (PMC10130558; doi:10.1111/eva.13531)
Supplement: Supplementary file 8 — Table S4. [file EVA-16-772-s008.docx]

**Table S4.** List of single nucleotid polymorphisms associated with the leaf consumption index. The 10 most associated SNPs are presented for each chromosome. P-values corresponded to SNPs association tests with the response variable. Significance threshold indicate if the p-values of SNP outreached Bonferonni correction at 0.05 or 0.1 threshold.

| **Chromosome** | **Position** | **P-Value** | **Gene** | **Significance threshold** |
| --- | --- | --- | --- | --- |
| Chr1 | 2804826 | 2.907e-07 | No Gene found |  |
| Chr1 | 23337319 | 1.454e-06 | No Gene found |  |
| Chr1 | 8702745 | 2.380e-06 | [AT1G24560](http://arabidopsis.org/servlets/TairObject?name=AT1G24560.1&type=gene) |  |
| Chr1 | 7694932 | 3.384e-06 | No Gene found |  |
| Chr1 | 9391745 | 3.891e-06 | [AT1G27045](http://arabidopsis.org/servlets/TairObject?name=AT1G27045.1&type=gene) |  |
| Chr1 | 3094702 | 5.638e-06 | [AT1G09560](http://arabidopsis.org/servlets/TairObject?name=AT1G09560.1&type=gene) |  |
| Chr1 | 3093634 | 5.638e-06 | No Gene found |  |
| Chr1 | 3811711 | 6.405e-06 | [AT1G11330](http://arabidopsis.org/servlets/TairObject?name=AT1G11330.1&type=gene) |  |
| Chr1 | 3809016 | 6.405e-06 | No Gene found |  |
| Chr1 | 3810242 | 6.405e-06 | [AT1G11330](http://arabidopsis.org/servlets/TairObject?name=AT1G11330.1&type=gene) |  |
| Chr2 | 17924337 | 3.164e-08 | [AT2G43110](http://arabidopsis.org/servlets/TairObject?name=AT2G43110.1&type=gene) | **< 0.05** |
| Chr2 | 12136810 | 2.907e-07 | No Gene found |  |
| Chr2 | 1684194 | 6.540e-06 | No Gene found |  |
| Chr2 | 15945466 | 6.768e-06 | [AT2G38090](http://arabidopsis.org/servlets/TairObject?name=AT2G38090.1&type=gene) |  |
| Chr2 | 965782 | 8.073e-06 | No Gene found |  |
| Chr2 | 11699573 | 1.507e-05 | [AT2G27350](http://arabidopsis.org/servlets/TairObject?name=AT2G27350.1&type=gene) |  |
| Chr2 | 11702546 | 1.507e-05 | [AT2G27350](http://arabidopsis.org/servlets/TairObject?name=AT2G27350.1&type=gene) |  |
| Chr2 | 12679459 | 1.884e-05 | [AT2G29660](http://arabidopsis.org/servlets/TairObject?name=AT2G29660.1&type=gene) |  |
| Chr2 | 12679443 | 1.884e-05 | [AT2G29660](http://arabidopsis.org/servlets/TairObject?name=AT2G29660.1&type=gene) |  |
| Chr2 | 12679434 | 1.884e-05 | [AT2G29660](http://arabidopsis.org/servlets/TairObject?name=AT2G29660.1&type=gene) |  |
| Chr3 | 807041 | 3.164e-08 | [AT3G03380](http://arabidopsis.org/servlets/TairObject?name=AT3G03380.1&type=gene) | **< 0.05** |
| Chr3 | 3100595 | 3.164e-08 | [AT3G10050](http://arabidopsis.org/servlets/TairObject?name=AT3G10050.1&type=gene) | **< 0.05** |
| Chr3 | 1997235 | 1.784e-06 | No Gene found |  |
| Chr3 | 20353154 | 2.492e-06 | No Gene found |  |
| Chr3 | 10664460 | 5.638e-06 | No Gene found |  |
| Chr3 | 10681969 | 5.638e-06 | No Gene found |  |
| Chr3 | 10675882 | 5.638e-06 | No Gene found |  |
| Chr3 | 10670264 | 5.638e-06 | [AT3G28455](http://arabidopsis.org/servlets/TairObject?name=AT3G28455.1&type=gene) |  |
| Chr3 | 22625120 | 5.638e-06 | [AT3G61130](http://arabidopsis.org/servlets/TairObject?name=AT3G61130.1&type=gene) |  |
| Chr3 | 10662076 | 5.638e-06 | [AT3G28430](http://arabidopsis.org/servlets/TairObject?name=AT3G28430.1&type=gene) |  |
| Chr4 | 16396976 | 2.907e-07 | No Gene found |  |
| Chr4 | 16397084 | 2.907e-07 | No Gene found |  |
| Chr4 | 16394270 | 2.907e-07 | No Gene found |  |
| Chr4 | 16394598 | 2.907e-07 | No Gene found |  |
| Chr4 | 6941771 | 5.472e-06 | No Gene found |  |
| Chr4 | 5125918 | 5.638e-06 | No Gene found |  |
| Chr4 | 2315323 | 5.638e-06 | No Gene found |  |
| Chr4 | 495758 | 5.638e-06 | [AT4G01160](http://arabidopsis.org/servlets/TairObject?name=AT4G01160.1&type=gene) |  |
| Chr4 | 497621 | 5.638e-06 | No Gene found |  |
| Chr4 | 498205 | 5.638e-06 | No Gene found |  |
| Chr5 | 1030484 | 3.164e-08 | No Gene found | **< 0.05** |
| Chr5 | 24914441 | 2.907e-07 | [AT5G62000](http://arabidopsis.org/servlets/TairObject?name=AT5G62000.1&type=gene) |  |
| Chr5 | 24912551 | 2.907e-07 | [AT5G62000](http://arabidopsis.org/servlets/TairObject?name=AT5G62000.1&type=gene) |  |
| Chr5 | 5481252 | 1.405e-06 | [AT5G16700](http://arabidopsis.org/servlets/TairObject?name=AT5G16700.1&type=gene) |  |
| Chr5 | 9574471 | 1.969e-06 | No Gene found |  |
| Chr5 | 1608450 | 2.492e-06 | No Gene found |  |
| Chr5 | 26764754 | 3.270e-06 | No Gene found |  |
| Chr5 | 2512190 | 3.270e-06 | [AT5G07860](http://arabidopsis.org/servlets/TairObject?name=AT5G07860.1&type=gene) |  |
| Chr5 | 24904200 | 3.319e-06 | No Gene found |  |
| Chr5 | 2510842 | 5.007e-06 | No Gene found |  |
