# Supplementary material for: Plant–herbivore interactions: Experimental demonstration of genetic variability in plant–plant signalling
Source: Evol Appl. 2023 Mar 29;16(4):772–80. doi: 10.1111/eva.13531 (PMC10130558; doi:10.1111/eva.13531)
Supplement: Supplementary file 9 — Table S5. [file EVA-16-772-s003.docx]

| Response variable | SNP position | minor allele | major allele | minor allele effect |
| --- | --- | --- | --- | --- |
| First choice | Chr2: 8535984 | G/G (6.2%) | T/T | more attractive |
|  | Chr4: 13591215 | A/A (5.3%) | T/T | more attractive |
| Proportion of time | Chr1: 8702745 | G/G (6.2%) | T/T | more attractive |
|  | Chr2: 15945466 | C/C (7.1%) | A/A | more attractive |
|  | Chr3: 21777184 | C/C (8.8%) | T/T | more attractive |
|  | Chr5: 12097495 | A/A (7.1%) | G/G | more attractive |
| Leaf consumption index | Chr2: 17924337 | T/T (7.1%) | A/A | more repellant |
|  | Chr3: 807041 | C/C (7.1%) | T/T | more repellant |
|  | Chr3: 3100595 | C/C (7.1%) | T/T | more repellant |
|  | Chr5: 1030484 | G/G (7.1%) | A/A | more repellant |

**Table S5.** Effects of minor alleles of significant SNPs on response variables. The percentage of accessions with the minor alleles are given.
