# Supplementary material for: Plant–herbivore interactions: Experimental demonstration of genetic variability in plant–plant signalling
Source: Evol Appl. 2023 Mar 29;16(4):772–80. doi: 10.1111/eva.13531 (PMC10130558; doi:10.1111/eva.13531)
Supplement: Supplementary file 10 — Appendix S1. [file EVA-16-772-s002.docx]

**Supporting Information 1. Attractivity of clipped plants.**

**Material&Methods.** Eleven natural accessions of the 1001Genomes stock were used in this complementary experiment. Twelve individuals per accessions were sown in February 2021 on peat soil (Neuhaus N2). Plants grown during 6 weeks. At this date, two plants were presented in arena to snails. Pairs were constituted of an undamaged plant and of a clipped plant. The two individuals of a pair were of the same age and of the same accession identity. Snails were filmed during one hour and three variables were measured: The first choice of the snail, the proportion of time spent on each plant and the biomass consumption index described in main text. Three binomial models were applied to these variables in order to estimate the effect of clipping on the response variables with the intercept of models, and the surface difference effect with the slope estimated by the models.


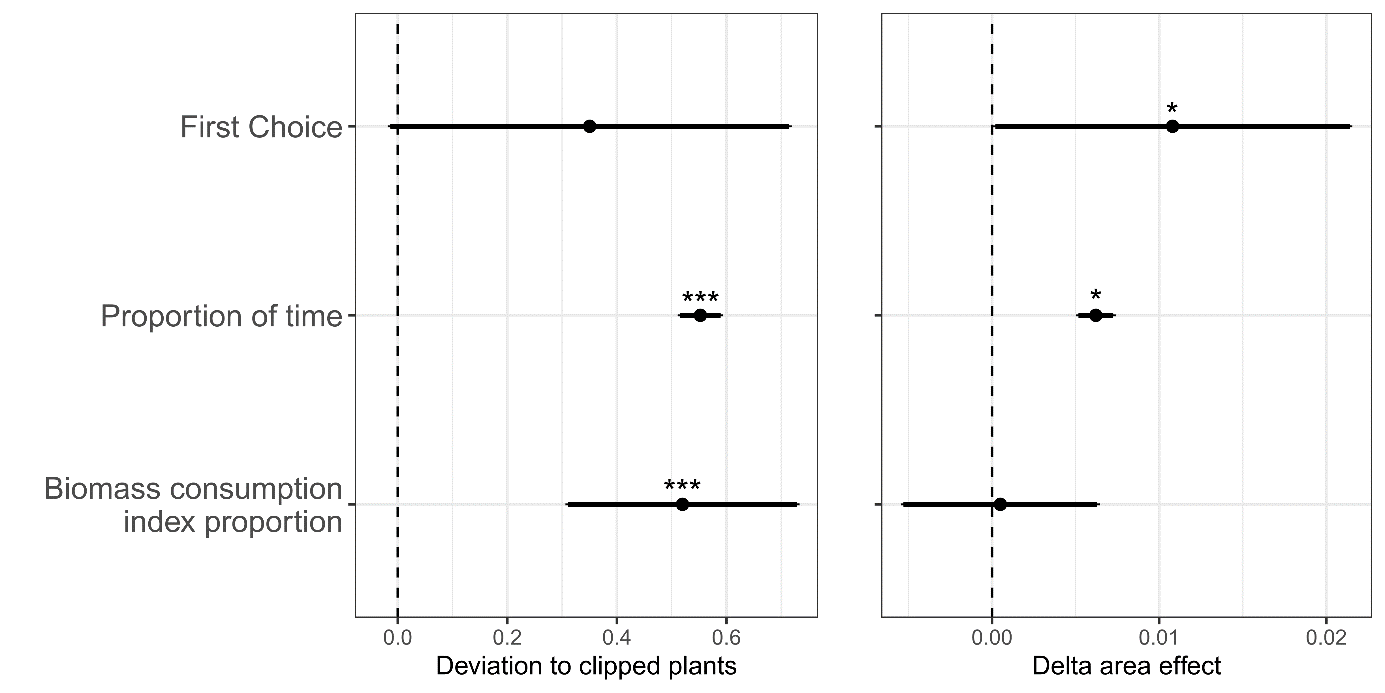
**Results.** In this experiment, clip damages did not have a significant effect on the first choice of snails (*P =* 0.054). Rosette area difference had a positive and slightly significant effect on the first choice of snails (*P* = 0.042). In other words, the bigger the plants, the bigger was the probability for the snail to choose it first. Clip damages had a significant and positive effect on the proportion of time spent on plants (*P* < 0.001). Snails spent significantly more time on clipped plants than on undamaged plants. Rosette area difference had a positive and significant effect on the proportion of time spent on plants (*P* < 0.001). Clip damages had a significant and positive effect on the leaf consumption index (*P* < 0.001) when rosette area difference did not have a significant effect on this response variable (*P* = 0.86).

**Figure SI 1.1.** Clipping and surface difference effects on response variables. Estimates of the coefficients of models are represented here on a probit scale. Horizontal bars represent standard errors of estimates multiplied by two. *: *P* < 0.05, **: *P* < 0.01; ***: *P* < 0.001
